# Academic Emotions in English-Medium Instruction: A Person-Centred Analysis of Emotional Profiles and Student Satisfaction

**DOI:** 10.3390/bs16060926

**Published:** 2026-06-05

**Authors:** Guadalupe de la Maya Retamar, Magdalena López-Pérez, Juan Luis de la Montaña Conchiña, José Luis Bravo Galán

**Affiliations:** 1 Department of Didactics of Social Sciences, Languages and Literatures, Faculty of Education and Psychology, University of Extremadura, 06006 Badajoz, Spain; magdalenalopez@unex.es (M.L.-P.); jmontana@unex.es (J.L.d.l.M.C.); 2 Department of Didactics of Experimental Sciences and Mathematic, Faculty of Education and Psychology, University of Extremadura, 06006 Badajoz, Spain; jlbravo@unex.es

**Keywords:** academic emotions, achievement emotion, English-medium instruction (EMI), emotional profiles, person-centred approach, student satisfaction

## Abstract

Academic emotions constitute a central component of students’ learning processes and overall academic satisfaction. Within English-Medium Instruction (EMI) contexts, learning through a foreign language may modulate students’ emotional experiences in complex ways. However, limited research has examined emotional profiles among students enrolled in EMI programmes. This study adopts a person-centred approach to identify emotional profiles based on students’ achievement emotions and to examine whether these profiles differ in terms of learning satisfaction. Participants were 128 undergraduate students enrolled in a bilingual degree programme at a Spanish university. Emotions were measured using the Achievement Emotions Questionnaire—Short Version (AEQ-S), and a k-means cluster analysis was conducted. The results revealed two distinct profiles: a more adaptive emotional profile, characterized by higher levels of enjoyment, hope, and pride, and a negative emotional profile, marked by higher levels of anger, anxiety, shame, hopelessness, and boredom. Students in the adaptive profile reported significantly higher levels of satisfaction, with a large effect size. No significant association was found between emotional profiles and students’ self-reported English proficiency, gender, and academic year. These findings suggest that fostering positive emotions—particularly enjoyment—and reducing deactivating negative emotions such as boredom and hopelessness may be key to enhancing student satisfaction in EMI programmes. Educators and institutions are encouraged to design emotionally supportive learning environments, going beyond a sole focus on language proficiency.

## 1. Introduction

Emotions are integral to teaching and learning processes. As Schutz and Lanehart (2002, p. 67) argue, “emotions are intimately involved in virtually every aspect of the teaching and learning process and, therefore, an understanding of the nature of emotions within the school context is essential.” Despite Pekrun’s (2024, p. 85) assertion that “the classroom is an emotional place,” the affective dimension of student learning and instructional practices has long remained underexplored within educational psychology and educational research. In recent decades, however, it has attracted increasing scholarly attention, particularly in the fields of academic emotions and second language learning (Pekrun, 2024; Dewaele & MacIntyre, 2016).

In educational settings, academic emotions—particularly achievement emotions—play a crucial role in students’ learning processes and academic experiences. These emotions, defined by Pekrun (2024) as emotions that occur in response to events and actions evaluated according to competence-based standards of quality, arise in achievement contexts such as studying, attending classes, or taking exams, and influence motivation, learning strategies, engagement, and academic performance (Pekrun et al., 2002). According to the Control–Value Theory of Achievement Emotions, students’ emotions are shaped by their perceptions of control over learning activities and the value they attribute to them (Pekrun et al., 2002; Pekrun, 2006).

In the 2000s, Pekrun and colleagues developed the Achievement Emotions Questionnaire (AEQ) (Pekrun et al., 2011), based on a bidimensional structure that classifies emotions by valence (pleasant vs. unpleasant) and level of physiological arousal (activation). More recently, Pekrun (2024) has proposed a revised conceptualization that not only refines this structure but also expands its scope, moving from an exclusive focus on achievement emotions to a more general theory of human emotions applicable across contexts. Within this framework, emotions can be organized through a three-dimensional taxonomy that considers several axes. First, concerning object focus and temporal dimension, emotions can be centred on the activity (the process of learning) or on the outcome (final success or failure) and may be situated in the past (retrospective), present (concurrent), or future (prospective). In academic settings, the most relevant combinations include enjoyment or boredom during the task (concurrent), hope and anxiety about the outcome (prospective), and pride or shame after the outcome (retrospective). Second, valence refers to whether the emotion is positive (pleasant, such as enjoyment) or negative (unpleasant, such as anxiety). Finally, activation classifies emotions according to the physiological arousal they produce, distinguishing between activating emotions (such as anger) and deactivating emotions (such as boredom).

Traditionally, research on academic emotions has mainly adopted variable-centred approaches, examining individual emotions separately. This body of work has provided valuable insights into the emotions that emerge in classroom settings and how specific emotions function in academic contexts (Robinson et al., 2017). However, students rarely experience emotions in isolation; instead, multiple emotions often co-occur during learning activities. For this reason, recent studies have increasingly adopted person-centred approaches, such as cluster analysis, to identify emotional profiles that capture patterns of co-occurring emotions among students and their influence on academic performance, engagement, and motivation (Ganotice et al., 2016; Robinson et al., 2017; Suriá-Martínez et al., 2025). These approaches provide a more comprehensive understanding of students’ emotional experiences.

The study of academic emotions may be particularly relevant in English-Medium Instruction (EMI) contexts. EMI refers to the use of English to teach academic subjects in countries where English is not the first language of most of the population (Dearden, 2014), as is the case in Spain. In this approach, the focus is not on the simultaneous learning of content and language, as occurs in Content and Language Integrated Learning (CLIL), but rather primarily on content learning, with language development often considered a secondary outcome (Su et al., 2021). As Macaro et al. (2018) point out, the intention to improve students’ English proficiency in EMI contexts is not always explicit or prioritised.

In recent years, EMI programmes have expanded rapidly across European universities, including Spain, as institutions seek to promote internationalisation and enhance students’ mobility, as well as their linguistic and professional competencies (Dang et al., 2023; Lasagabaster, 2022; Macaro et al., 2018). In this context, processes of globalisation and internationalisation have led to the implementation of foreign-language study programmes across Europe and worldwide (Doiz et al., 2013), resulting in what Coleman (2006) describes as the “Englishization” of European higher education. The objectives are twofold: on the one hand, to prepare students to participate and compete in an increasingly globalised world; on the other, to enhance universities’ visibility, improve their position in international rankings, and increase their competitiveness. However, internationalisation in higher education is not merely an administrative strategy but an intentional process aimed at integrating international and intercultural dimensions into teaching, research, and institutional practices (Bowles & Murphy, 2020).

Previous research on EMI has largely focused on its implementation in higher education and the pedagogical challenges associated with teaching disciplinary content through English (Doiz et al., 2013; Dang et al., 2023; Shao & Rose, 2024; Yuan et al., 2022; Yuan & Yang, 2023). Within this body of literature, several factors have been identified as shaping students’ performance in both language development and disciplinary learning. Guo et al. (2022), for example, distinguish between individual factors—such as prior knowledge, effort, and interest—and contextual factors related to the learning environment, including course design, teaching practices, and the availability of learning resources.

Evidence regarding the outcomes of EMI implementation remains inconclusive. While some studies report positive effects on students’ English proficiency (Hernández-Nanclares & Jiménez-Muñoz, 2017; Richter, 2019), these gains may be smaller than expected (Vidal & Jarvis, 2020). In addition, research suggests that studying through English does not necessarily hinder the acquisition of disciplinary knowledge (Dafouz & Camacho-Miñano, 2016; Lasagabaster, 2022). In contrast, other studies highlight challenges that may affect students’ understanding of content and their attitudes towards EMI (Hengsadeekul et al., 2014), as well as the frequent reliance on the first language (L1) and the potential simplification of academic content in EMI contexts (Beckett & Li, 2012). Scholars have also pointed out that the widespread adoption of EMI may generate tensions between the growing use of English and the preservation of national languages and academic cultures (Bowles & Murphy, 2020; Macedo et al., 2015).

More recent reviews likewise indicate that findings on both language development and content learning remain mixed and highly context-dependent (Curle et al., 2025; Rose et al., 2020). Therefore, the available evidence remains insufficient to conclusively determine whether EMI consistently benefits language learning or whether it may entail long-term costs for the deep understanding of complex content (Macaro et al., 2018; Yuan et al., 2023a). Moreover, further experimental or quasi-experimental research is needed, based on more representative samples and including cross-institutional analyses (Fernández Costales & Lasagabaster Herrarte, 2024). In addition, there is a need for more precise assessment instruments, as Curle et al. (2025) argue that standardized tests do not capture the differential effects of EMI.

However, EMI should also be understood as a multidimensional educational experience (Yuan et al., 2023a). From this perspective, learning through a foreign language may introduce additional cognitive, social, and emotional challenges for students. Previous research suggests that EMI settings may be associated with emotional experiences such as anxiety, frustration, or decreased confidence, particularly when students struggle to understand content delivered in English (Macaro et al., 2018). At the same time, EMI can foster positive emotions, such as pride and enjoyment, when students perceive gains in language proficiency and academic success.

Despite the growing interest in EMI and the increasing recognition of the role of affective factors in learning, these dimensions have hitherto been largely overlooked (Lasagabaster et al., 2024), and relatively little research has examined how different academic emotions combine to shape students’ emotional experiences in EMI settings. Most previous studies have focused on individual emotions in isolation, typically using variable-centred approaches (Dai & Wang, 2024; Hopkyns & Gkonou, 2023; Kopinska & Fernández-Costales, 2023; Liyanage, 2023; Şahan & Sahan, 2023; Xu et al., 2025; Yuan et al., 2023a, 2023b), with particular attention given to anxiety, especially in relation to oral performance, exams, and communication apprehension. In addition to anxiety, other negative emotions that have been examined include frustration, disappointment, shame, boredom, guilt, and fear. Among positive emotions, studies have highlighted the role of pride, hope, enthusiasm, confidence, and enjoyment.

Students, however, tend to experience multiple emotions simultaneously during learning activities, particularly in demanding contexts such as EMI, where disciplinary content must be processed in a second language. In this regard, person-centred approaches may provide a more comprehensive understanding of students’ emotional experiences in EMI learning environments. Nevertheless, research adopting this perspective in EMI contexts remains scarce. Most existing studies have examined individual emotions in isolation, focusing predominantly on anxiety and, to a lesser extent, on enjoyment or boredom. As a result, little is known about how multiple emotions co-occur and interact to shape students’ overall affective experience in EMI settings or how these combined emotional patterns relate to outcomes such as learning satisfaction.

For the purposes of this study, emotional profiles are understood as distinct patterns of co-occurring academic emotions that characterize groups of students, as identified through person-centred statistical approaches (Robinson et al., 2017). Student satisfaction, in turn, refers to students’ overall subjective evaluation of their learning experience in EMI courses, encompassing their perceived quality of instruction, engagement, and well-being within the programme (cf. Kopinska & Fernández-Costales, 2023; Tsui & Cheng, 2022).

Furthermore, as it has been previously mentioned, students’ emotional experiences may influence their satisfaction with EMI courses. Students who experience predominantly positive emotions are more likely to perceive EMI learning environments positively (Kopinska & Fernández-Costales, 2023), whereas those experiencing negative emotions may report lower levels of satisfaction (Tsui & Cheng, 2022).

Therefore, the present study aims to identify emotional profiles based on students’ academic emotions in an EMI context and to examine whether these profiles differ in terms of satisfaction. To address this objective, the following research questions are formulated:

RQ1: What emotional profiles can be identified among students enrolled in EMI courses based on their academic emotions?

RQ2: Do the identified emotional profiles differ in terms of students’ satisfaction with their learning experience?

RQ3: Is there an association between students’ English proficiency levels, gender, and academic year and their emotional profiles?

## 2. Materials and Methods

This study employed a cross-sectional survey design. Data were collected at a single point in time from undergraduate students enrolled in an EMI programme, with the aim of identifying emotional profiles and examining their relationship with learning satisfaction, English proficiency, gender, and academic year.

### 2.1. Participants

The sample consisted of 128 undergraduate students from the University of Extremadura, all enrolled in a Social Sciences degree programme delivered in a bilingual format, in which approximately 50% of the courses are taught in English. Although participants were selected through convenience sampling, as access was determined by institutional availability, data were gathered from all students present in the classroom on the day of administration across the three available year groups; fourth-year students were not included, as they were engaged in compulsory teaching placements during the data collection period. This fact strengthens the representativeness of the sample within this specific programme and context. Participants ranged in age from 18 to 30 years (M = 19.95, SD = 1.87). In terms of gender distribution, 81 identified as female (63.3%), 46 as male (35.9%), and 1 participant (0.8%) preferred not to disclose their gender. Regarding self-reported English proficiency, the majority of participants reported CEFR levels B1 (n = 54, 42.5%) and B2 (n = 45, 35.4%), followed by A2 (n = 11, 8.7%) and C1 (n = 8, 6.3%). Eight participants (6.3%) did not indicate their proficiency level. The distribution across the academic year was as follows: 55 first-year students (43.0%), 33 second-year students (25.8%), and 40 third-year students (31.3%).

No formal power analysis was conducted prior to data collection, as the sample comprised all available students within the programme. Nevertheless, the total sample size of 128 participants is consistent with recommendations for k-means cluster analysis, which generally require a minimum of 50 cases per cluster to obtain stable solutions (Hair et al., 2019), a threshold exceeded by both clusters obtained in this study.

### 2.2. Instruments

Academic emotions were assessed using the Achievement Emotions Questionnaire—Short Version (AEQ-S; Bieleke et al., 2021), an abbreviated form of the original Achievement Emotions Questionnaire (Pekrun et al., 2011). The AEQ is a self-report instrument designed to measure students’ achievement-related emotions, including enjoyment, hope, pride, anger, anxiety, shame, hopelessness, and boredom.

Given the considerable length of the original AEQ (232 items across 24 scales) and the associated administration time, Bieleke et al. (2021) developed the AEQ-S to provide a more efficient alternative while preserving the conceptual integrity of the original instrument. The short version comprises four items per scale and has demonstrated satisfactory psychometric properties. In the present study, internal consistency coefficients were acceptable to high across all scales (α = 0.75 for enjoyment; α = 0.70 for hope; α = 0.70 for pride; α = 0.71 for anger; α = 0.77 for anxiety; α = 0.86 for shame; α = 0.80 for hopelessness; and α = 0.90 for boredom).

Although the AEQ assesses emotions across three academic contexts (attending class, studying, and test-taking) and three temporal phases (before, during, and after), the present study focused exclusively on emotions experienced during class time. Accordingly, participants completed 32 items, randomly ordered, with four items corresponding to each of the eight emotions assessed. Responses were recorded on a five-point Likert scale ranging from 1 (strongly disagree) to 5 (strongly agree).

To complement the AEQ-S data, additional items were included to explore students’ emotional experiences in greater depth. These considered both open- and closed-ended questions addressing the perceived causes of emotions and their evolution over time.

Student satisfaction with the EMI learning experience was assessed using a single item: ‘Rate your level of satisfaction with this English-medium course’, with responses recorded on a ten-point scale ranging from 1 (not at all satisfied) to 10 (very satisfied). The item was thus specifically designed to capture students’ overall evaluation of the course as delivered through English, rather than their satisfaction with the subject matter itself. Although multi-item scales are generally preferred, single-item measures have been shown to provide adequate reliability and validity for concrete, unidimensional constructs such as overall satisfaction (Nagy, 2002; Wanous & Hudy, 2001).

Finally, participants completed a sociolinguistic questionnaire designed to collect demographic information, details about their language learning background, and relevant characteristics of the academic programme. Self-reported English proficiency was assessed through a single item included in the sociolinguistic questionnaire, in which participants were asked to indicate their current level of English according to the Common European Framework of Reference for Languages (CEFR). Response options were presented as an ordinal scale ranging from A1 (beginner) to C2 (proficient). Although objective measures such as standardized language tests or academic grades might provide a more reliable indicator of proficiency, self-reported CEFR levels were considered appropriate for the purposes of this study, given its focus on students’ subjective perceptions of their learning experience. Furthermore, academic grades reflect a broader range of factors beyond language proficiency and were therefore not considered a suitable proxy.

### 2.3. Procedure

Data were collected through the administration of a questionnaire to undergraduate students enrolled in different years of the degree programme. Prior to data collection, participants were informed about the aims of the study, the procedures involved, and the confidentiality of their responses. All participants provided informed consent and agreed to take part voluntarily and anonymously.

Following consent, the participants completed the questionnaire in Spanish using an online platform. The survey required approximately 10 min to complete. During administration, at least one member of the research team was present in the classroom to provide clarification and ensure standardised conditions across groups.

Data collection took place in March 2023, approximately eight weeks after the beginning of the academic term. The timing ensured that all participants had sufficient exposure to English-medium instruction (EMI) in their courses to report meaningful emotional experiences.

The study was conducted in accordance with the principles of the Declaration of Helsinki and received ethical approval from the Ethics Committee of the University of Extremadura.

### 2.4. Data Analysis

Following data collection, the dataset was processed and analysed using IBM SPSS (Version 23). Prior to the main analyses, missing data were checked and found to be minimal. Homogeneity of variances was assessed using Levene’s test prior to each between-group comparison, and non-parametric alternatives were applied where this assumption was violated. Outlier screening using z-scores revealed extreme values in two cases, all falling within the valid range of the scale and reflecting internally consistent response patterns. These cases were therefore retained in the analyses. Cluster membership served as the grouping variable in the comparative analyses, student satisfaction as the dependent variable, and English proficiency, gender, and academic year as additional grouping variables in the association analysis. Descriptive statistics (means and standard deviations) were first computed for all study variables.

To identify patterns of co-occurring academic emotions, a k-means cluster analysis was performed using the eight emotional variables. This person-centred approach enabled the identification of distinct emotional profiles based on students’ combined emotional experiences.

K-means cluster analysis was selected because it is well-suited to identifying distinct groups of participants based on continuous variables and has been widely used in person-centred research on emotional profiles in educational settings (Ganotice et al., 2016; Robinson et al., 2017; Suriá-Martínez et al., 2025). Unlike hierarchical methods, k-means is particularly appropriate for larger samples and produces stable and interpretable solutions. The number of clusters was determined by testing solutions ranging from two to five clusters and evaluating each solution based on conceptual interpretability, the relative size of the resulting groups, and the degree of differentiation between clusters across all emotional variables. The two-cluster solution was retained as it produced the most theoretically meaningful and balanced partition of the data, with clearly contrasting emotional profiles and a near-equal distribution of cases (n = 65 and n = 63). Internal validation was further supported by the significant differences observed between clusters across all eight emotions, as reported in Section 3.2.

Differences between the resulting clusters were examined using independent-samples *t*-tests. When the assumption of homogeneity of variances was violated, Mann–Whitney U tests were employed as a non-parametric alternative. A one-way ANOVA was also conducted to examine whether satisfaction differed across the academic year. To control the increased risk of Type I error due to multiple comparisons, a Bonferroni correction was applied, setting the adjusted significance threshold at *p* < 0.006 (α = 0.05/8). All comparisons remained statistically significant after correction.

Students’ satisfaction levels were compared across emotional profiles using an independent-samples *t*-test. Additionally, a Mann-Whitney U test was conducted to examine differences in self-reported English proficiency between emotional profiles, given the ordinal nature of the proficiency measure. Chi-square tests of independence were also conducted to explore the association between emotional profiles and gender and between emotional profiles and academic year.

## 3. Results

### 3.1. Descriptive Statistics and Correlations

Prior to the main analyses, descriptive statistics and intercorrelations among the study variables were examined. As shown in Table 1, among the positive emotions, pride showed the highest mean score (M = 3.65, SD = 0.73), followed by hope (M = 3.48, SD = 0.78) and enjoyment (M = 3.08, SD = 0.80). Regarding negative emotions, boredom obtained the highest mean (M = 2.82, SD = 1.12), followed by shame (M = 2.47, SD = 1.07), anxiety (M = 2.25, SD = 0.90), and anger (M = 2.10, SD = 0.83). Hopelessness showed the lowest mean score of all emotions (M = 1.80, SD = 0.83).

Regarding student satisfaction with the EMI learning experience, the mean score was 6.49 (SD = 1.96) on a ten-point scale, with scores ranging from 1 to 10, indicating a moderate-to-positive overall level of satisfaction among participants.

Table 2 displays the intercorrelation matrix. Positive emotions were moderately to strongly correlated with each other (r = 0.71–0.74, *p* < 0.01) and negatively correlated with all negative emotions. Among negative emotions, anxiety and shame showed the strongest intercorrelation (r = 0.79, *p* < 0.01), followed by anger and hopelessness (r = 0.74, *p* < 0.01). Enjoyment showed the strongest negative correlation with boredom (r = −0.68, *p* < 0.01). All emotions were significantly correlated with satisfaction in the expected direction, with hope (r = 0.63, *p* < 0.01) and enjoyment (r = 0.60, *p* < 0.01) showing the strongest positive associations, and hopelessness (r = −0.56, *p* < 0.01) and boredom (r = −0.51, *p* < 0.01) the strongest negative ones.

### 3.2. Emotional Profiles

A k-means cluster analysis was conducted using students’ academic emotions (enjoyment, hope, pride, anger, anxiety, shame, hopelessness, and boredom), as measured by the AEQ-S questionnaire. The analysis yielded a two-cluster solution comprising 128 participants: Cluster 1 (n = 65) and Cluster 2 (n = 63). The clusters were relatively balanced in size. The two-cluster solution was retained based on its conceptual interpretability and the balanced distribution of cases across clusters, which are considered key criteria in cluster validation.

### 3.3. Differences in Academic Emotions Across Profiles

**Figure 1:**
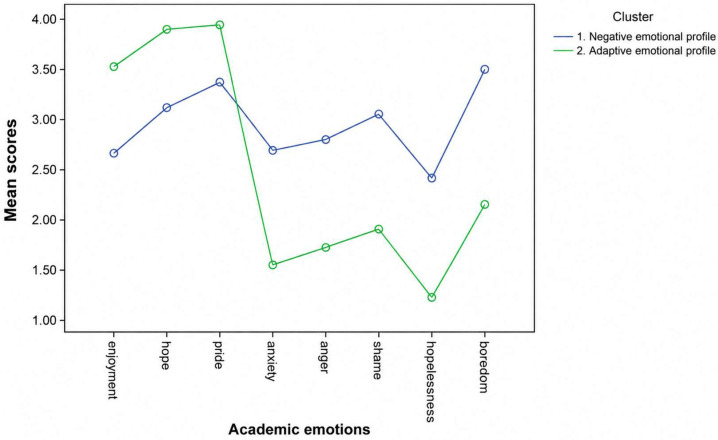
Mean scores of academic emotions across emotional profiles.

The cluster solution identified in the analysis presented in Section 3.2 revealed distinct emotional patterns across groups. Table 3 presents the mean scores for each academic emotion by cluster, while Figure 1 provides a graphical representation of these differences.

Cluster 1 was characterized by lower levels of positive emotions (enjoyment, hope, and pride) and higher levels of negative emotions, including anger, anxiety, shame, boredom, and hopelessness. This pattern is indicative of a predominantly negative emotional profile.

In contrast, Cluster 2 displayed higher levels of positive emotions and lower levels of negative emotions, particularly boredom and hopelessness, reflecting a more adaptive emotional profile.

To examine differences between clusters, independent-samples *t*-tests were conducted for variables meeting the assumption of homogeneity of variances. For anxiety, shame, and hopelessness, where this assumption was violated, Mann–Whitney U tests were employed as a non-parametric alternative.

The results indicated statistically significant differences between clusters across all emotions: enjoyment (*t* = 6.96, *p* < 0.001), hope (*t* = 6.79, *p* < 0.001), pride (*t* = 4.98, *p* < 0.001), anger (*t* = 10.87, *p* < 0.001), and boredom (*t* = 8.53, *p* < 0.001), as well as anxiety (*Z* = −7.18, *p* < 0.001), shame (*Z* = −6.21, *p* < 0.001), and hopelessness (*Z* = −8.74, *p* < 0.001). All differences remained statistically significant after applying Bonferroni correction (adjusted α = 0.006).

### 3.4. Differences in Satisfaction Across Emotional Profiles

Differences in students’ satisfaction across emotional profiles were examined using an independent-samples *t*-test. As the assumption of homogeneity of variances was met, equal variances were assumed. The results revealed statistically significant differences between the two profiles, with students in the adaptive emotional profile reporting higher levels of satisfaction than those in the negative emotional profile (*t* = 6.96, *p* < 0.001). The mean difference between groups was 2.06, with a 95% confidence interval ranging from 1.48 to 2.65. The effect size was large (Cohen’s *d* = 1.23), indicating a substantial difference between groups. A one-way ANOVA revealed no significant differences in satisfaction across academic years (F = 1.62, *p* = 0.202), suggesting that satisfaction levels were comparable across cohorts.

### 3.5. English Proficiency and Emotional Profiles

A Mann–Whitney U test was conducted to examine differences in self-reported English proficiency between emotional profiles, given the ordinal nature of the proficiency measure. It should be noted that English proficiency was assessed through students’ self-perception rather than through a standardised or objective measure. The results did not reveal a statistically significant difference between profiles (U = 1560, *p* = 0.262), suggesting that emotional profiles were not associated with students’ perceived levels of English proficiency.

### 3.6. Gender, Academic Year, and Emotional Profiles

Chi-square tests of independence revealed no significant association between emotional profiles and gender (χ^2^ = 1.30, df = 2, *p* = 0.521), nor between emotional profiles and academic year (χ^2^ = 3.57, df = 2, *p* = 0.168). These results suggest that the emotional profiles identified in this study are not explained by gender or cohort differences. It should be noted that some expected cell frequencies in the gender analysis were below the recommended threshold due to the small number of participants who preferred not to disclose their gender. Therefore, these results should be interpreted with caution.

## 4. Discussion

Research on academic emotions has increasingly emphasized the central role of affective processes in learning, particularly in contexts involving additional cognitive demands. In English-Medium Instruction (EMI) settings, students must process disciplinary content through a foreign language, which can intensify both positive and negative emotional experiences. Although interest in emotions in EMI has grown in recent years, the field remains underdeveloped, with a predominant focus on individual emotions and relatively limited use of person-centred approaches to capture the complexity of students’ emotional experiences. As a result, direct comparisons with previous findings remain constrained.

The present study sought to identify emotional profiles among students enrolled in an English-Medium Instruction (EMI) by adopting a person-centred approach to examine how multiple academic emotions co-occur and combine to shape students’ affective experiences and to explore whether the resulting emotional profiles differ in terms of learning satisfaction and English proficiency. Most previous research in this field has focused on individual emotions in isolation, particularly anxiety, with relatively little attention paid to the combined emotional patterns characterizing different groups of students. The results revealed two distinct emotional profiles characterized by contrasting configurations of positive and negative academic emotions, thereby providing a more integrated understanding of the affective dimension of EMI learning.

### 4.1. Emotional Profiles in EMI

The two profiles identified in this study reflect a clear contrast between adaptive and maladaptive emotional configurations. While differences between profiles were significant across all eight emotions, the gap was particularly pronounced for negative deactivating emotions: boredom and hopelessness showed the largest between-cluster differences, with very low values in the adaptive profile and markedly higher values in the negative one. This pattern suggests that what most distinguishes students in the negative profile is not simply the presence of negative emotions, but specifically the predominance of disengaging experiences that undermine motivation and persistence.

Regarding negative emotions, the gap between profiles was considerably larger, particularly for boredom, which showed notably high values in the negative profile, as well as for shame. A more fine-grained analysis of item-level responses further supports this pattern, showing that boredom and hopelessness consistently differentiate the two profiles, with very low levels in the adaptive profile and markedly higher values in the negative profile. These findings suggest that the distinction between profiles is not only characterized by lower levels of positive emotions but also by the presence of disengaging emotional experiences.

These results are consistent with the Control–Value Theory of Achievement Emotions (Pekrun, 2006), which posits that students’ emotional experiences are shaped by their perceived control over learning activities and the value they attribute to these learning situations. From this perspective, the two profiles identified in this study may reflect underlying differences in these appraisals. Students in the negative emotional profile are likely to experience lower perceived control and task value, whereas students in the more adaptive emotional profile may benefit from favourable motivational conditions, leading to higher motivation, greater persistence, and better academic outcomes.

The findings also reinforce previous research suggesting that academic emotions tend to co-occur in meaningful patterns rather than being experienced in isolation (Ganotice et al., 2016; Robinson et al., 2017). Within EMI settings, the presence of mixed and fluctuating emotional experiences among students is relatively common, often resulting in what has been described as an “emotional rollercoaster” (Hopkyns & Gkonou, 2023). The present findings extend this line of research by demonstrating that these co-occurring emotions can be meaningfully grouped into distinct profiles with differential implications.

All the emotions identified in this study have also been reported in previous research. For instance, enjoyment has been described as a key driver of academic engagement and willingness to communicate (Dai & Wang, 2024; Xu et al., 2025). Some students have even compared EMI to a durian fruit: initially intimidating and challenging but ultimately rewarding as confidence and knowledge develop over time (Yuan et al., 2023b). Hope and pride are often linked to broader expectations of global mobility and academic success (Hillman et al., 2023; Yuan et al., 2023b), with hope reflecting students’ initial expectations and pride emerging from overcoming linguistic challenges or being part of prestigious programmes (Şahan & Sahan, 2023).

Negative emotions are also widely documented in the literature. Shame, in particular, has been identified as a salient emotion, often functioning as a mechanism of social and personal regulation (Liyanage, 2023), and typically arising when students perceive themselves as having lower levels of linguistic competence or when comparing themselves with more fluent peers (Şahan & Sahan, 2023). Similarly, boredom has been associated with monologic and monotonous teaching practices (Dai & Wang, 2024), where limited interaction may lead to reduced attention and motivation (Kopinska & Fernández-Costales, 2023), as well as with students’ disengagement when they struggle to understand abstract concepts in English (Yuan et al., 2023a).

Notably, in the present study, nearly half of the students were classified within the negative emotional profile. In this regard, the concept of emotional insecurity (Méndez Santos, 2020) is particularly relevant, as it has been identified as a key factor underlying both amotivation—such as the decision not to enrol in EMI courses—and demotivation, reflected in a loss of interest during the course. This perspective is complemented by Şahan and Sahan (2023), who refer to the notion of “emotional damage.” In both cases, these emotional experiences are not merely transient states but rather structural psychological barriers within EMI contexts, arising from the dual pressure of mastering both content and language. Without adequate affective support, such experiences may ultimately lead to student disengagement, isolation, or even withdrawal.

Furthermore, the emotional profiles identified in this study were not significantly associated with gender or academic year, suggesting that the two configurations of emotional experience observed are not merely a reflection of demographic characteristics. This finding adds robustness to the profiles as meaningful psychological constructs cutting across student subgroups.

### 4.2. Emotional Profiles and Student Satisfaction

In line with the emotional patterns described above, one of the most relevant findings of this study is the strong association between emotional profiles and students’ satisfaction. Students in the adaptive emotional profile reported significantly higher levels of satisfaction than those in the negative emotional profile, with a large effect size. This finding highlights the central role of emotions in shaping students’ perceptions of their learning experience, particularly in demanding contexts such as EMI, and reinforces the importance of considering students’ emotional experiences as a key component of quality in EMI programmes.

In EMI settings, where students are required to process academic content through a foreign language, emotional experiences may be particularly salient. The additional cognitive demands associated with learning in a second language may intensify both positive and negative emotional responses, with the latter often leading to stress, fatigue, and fear of failure (Hopkyns & Gkonou, 2023; Kopinska & Fernández-Costales, 2023). Students who experience higher levels of enjoyment and lower levels of boredom and hopelessness are more likely to engage with the learning process (Dai & Wang, 2024), which in turn may enhance their overall satisfaction.

Several studies have examined the relationship between students’ satisfaction and EMI programmes, linking it to their emotional experiences. For instance, Kopinska and Fernández-Costales (2023) associate positive evaluations of the learning experience, among other factors, with a low-anxiety classroom atmosphere in EMI contexts, which in turn enhances students’ motivation to engage in this type of instruction. Yuan et al. (2023a) document emotional changes and increased satisfaction associated with more reflective and participatory teaching methods.

Although the study by Dai and Wang (2024) focuses on academic engagement, it employs structural equation modelling (SEM) to demonstrate that positive emotions, such as enjoyment, predict higher levels of engagement and more positive interaction within EMI contexts. However, just as positive emotions are associated with higher satisfaction, some studies also report student dissatisfaction linked to negative emotional experiences. Tsui and Cheng (2022) refer to research showing high levels of dissatisfaction with EMI content instruction, even among students with high levels of language proficiency, due to excessive anxiety and negative perceptions of the course.

### 4.3. Emotional Profiles and English Proficiency

Contrary to expectations, no significant association was found between students’ self-reported English proficiency and emotional profiles. This finding contrasts with previous research suggesting that language proficiency not only shapes students’ emotional experiences but that emotions also act as mediators in their performance and learning (Méndez Santos, 2020; Şahan & Sahan, 2023; Xu et al., 2025). However, the present results suggest that emotional experiences in EMI may not depend solely on language proficiency. Other factors, such as teaching practices, task design, or students’ motivational beliefs, may play a more relevant role. This result should be interpreted with caution, as English proficiency was assessed through self-report rather than an objective measure. Furthermore, it should be noted that the present study adopts a person-centred approach based on emotional profiles, which focuses on patterns of co-occurring emotions rather than individual variables, and may therefore partly explain the absence of a direct association with English proficiency.

These findings have important implications for EMI programmes. Rather than focusing exclusively on students’ language proficiency, educators should also consider the emotional dimension of learning. Promoting positive emotions such as enjoyment and reducing negative emotions such as anxiety and boredom may contribute to more effective and satisfying learning experiences. This could be achieved using supportive teaching practices, clear instructional design, and opportunities for active participation. As argued by Hillman et al. (2023), it is essential to foster an emotionally supportive environment in EMI contexts—that is, an educational space that not only focuses on the transmission of academic content but also actively recognizes and validates the affective dimension of all participants, including students, teachers, and administrators. Such an approach may help reduce emotional barriers and enhance both student engagement and overall learning outcomes in EMI programmes.

This study has several limitations that should be considered when interpreting the findings. Firstly, the sample was drawn from a single bilingual programme at one Spanish university, which limits the generalizability of the results to other EMI contexts, institutions, or student populations. Given that emotional experiences in EMI are inherently context-dependent, the two profiles identified here may not be representative of students in programmes with different institutional structures, language policies, or pedagogical approaches. Secondly, all emotional data were collected by means of self-report measures, which are susceptible to social desirability bias and retrospective distortion. Students’ responses may not fully capture the complexity and fluctuation of their emotional experiences during EMI classes. Thirdly, English proficiency was assessed through self-perception rather than through a standardized or objective measure, which may introduce response bias and limit the validity of the proficiency-related findings. Future studies should address these limitations by including larger and more diverse samples across different EMI contexts, incorporating objective assessments of language proficiency, and considering longitudinal designs capturing the evolution of students’ emotional experiences over time.

## 5. Conclusions

This study contributes to the growing body of research on academic emotions in EMI by demonstrating that students’ affective experiences in these contexts are not adequately captured by examining individual emotions in isolation. By adopting a person-centred approach, we identified two distinct emotional profiles reflecting meaningful differences in how students experience EMI learning, with the negative profile characterised above all by the predominance of disengaging emotions such as boredom and hopelessness. The strong association between emotional profiles and satisfaction underscores the centrality of the affective dimension in students’ overall evaluation of EMI programmes, and suggests that emotional experience may be a more powerful predictor of satisfaction than language proficiency alone.

These findings have direct implications for the design of EMI programmes. Rather than focusing exclusively on linguistic outcomes, educators and institutions should consider how instructional practices, task design, and classroom climate shape students’ emotional experiences. Creating conditions that reduce disengagement and foster enjoyment may prove as important for student success as addressing language barriers. Future research should build on these findings through longitudinal and cross-institutional designs that capture how emotional profiles evolve over time and vary across different EMI contexts.

## Figures and Tables

**Table 1:** Descriptive statistics.

	n	Min	Max	M	SD
Enjoyment	128	1	5	3.08	0.80
Hope	128	1	5	3.48	0.78
Pride	128	1	5	3.65	0.73
Anger	128	1	5	2.10	0.83
Anxiety	128	1	5	2.25	0.90
Shame	128	1	5	2.47	1.07
Hopelessness	128	1	5	1.80	0.83
Boredom	128	1	5	2.82	1.12

**Table 2:** Descriptive statistics and intercorrelations among study variables (n = 128).

Variable	1	2	3	4	5	6	7	8	9
1. Enjoyment	-								
2. Hope	0.74 **	-							
3. Pride	0.70 **	0.70 **	-						
4. Anger	−0.46 **	−0.36 **	−0.34 **	-					
5. Anxiety	−0.33 **	−0.39 **	−0.31 **	0.51 **	-				
6. Shame	−0.28 **	−0.32 **	−0.21 *	0.36 **	0.79 **	-			
7. Hopelessness	−0.46 **	−0.48 **	−0.46 **	0.74 **	0.63 **	0.52 **	-		
8. Boredom	−0.69 **	−0.45 **	−0.36 **	0.65 **	0.28 **	0.24 **	0.49 **	-	
9. Satisfaction	0.60 **	0.62 **	0.58 **	−0.44 **	−0.40 **	−0.26 **	−0.55 **	−0.51 **	-

* *p* < 0.05. ** *p* < 0.01.

**Table 3:** Mean scores of academic emotions by cluster.

Emotion	Cluster 1	Cluster 2
Enjoyment	2.65 (±0.62)	3.52 (±0.71)
Hope	3.08 (±0.68)	3.89 (±0.65)
Pride	3.36 (±0.63)	3.95 (±0.70)
Anger	2.67 (±0.70)	1.52 (±0.48)
Anxiety	2.79 (±0.85)	1.70 (±0.50)
Shame	3.04 (±1.02)	1.88 (±0.75)
Hopelessness	2.40 (±0.74)	1.19 (±0.32)
Boredom	3.49 (±0.81)	2.13 (±0.99)

## Data Availability

The dataset is available upon request from the authors.

## Abbreviations

The following abbreviations are used in this manuscript:

CLIL	Content and Language Integrated Learning
EMI	English-Medium Instruction
